# The PXR is a drug target for chronic inflammatory liver disease^☆^

**DOI:** 10.1016/j.jsbmb.2010.04.012

**Published:** 2010-05-31

**Authors:** Karen Wallace, David E. Cowie, Dimitrios K. Konstantinou, Stephen J. Hill, Torunn E. Tjelle, Andrew Axon, Matthew Koruth, Steven A. White, Harald Carlsen, Derek A. Mann, Matthew C. Wright

**Affiliations:** a Institute of Cellular Medicine, University of Newcastle, Newcastle, Upon Tyne, UK; b Institute Medical Sciences, University of Aberdeen, Aberdeen, UK; c Department of Nutrition, University of Oslo, Norway

**Keywords:** ALT, alanine aminotransferase, CsA, cyclosporin A, GT, gliotoxin, GAPDH, glyceradehyde 3 phosphate dehydrogenase, HYP, hyperforin, IKK2-In, IκB kinase 2 inhibitor, LPS, lipopolysaccharide, METYR, metyrapone, MTS, ([3-(4,5-dimethylthiazol-2-yl)-5-(3-carboxymethoxyphenyl)-2-(4-sulfophenyl)-2H-tetrazolium salt, PBC, primary biliary cirrhosis, PCN, pregnenolone 16α carbonitrile, PTI, portal tract inflammation, PPARγ, peroxiome proliferator activated receptor γ, PXR, pregnane X receptor, RIF, rifampicin, SULF, sulfasalazine, TLR4, toll-like receptor 4, TNFα, tumour necrosis factor-α, Pregnane X receptor, SXR, NF-κB, Rifampicin, Hyperforin, TNFα

## Abstract

PXR activators are used to treat pruritus in chronic inflammatory liver diseases such as primary biliary cirrhosis (PBC). The aims of this study were to determine whether PXR activators could have an additional benefit of inhibiting inflammation in the liver, and determine whether cyclosporin A – which more effectively prevents PBC recurrence in transplanted patients than FK506 – is a PXR activator. In SJL/J mice (which have constitutively high levels of hepatic portal tract inflammatory cell recruitment), feeding a PXR activator inhibited inflammation, TNFα and Il-1α mRNA expression in SJL/J-PXR^+/+^, but not SJL/J-PXR^−/−^. Monocytic cells – a major source of inflammatory mediators such as TNFα – expressed the PXR and PXR activators inhibited endotoxin-induced NF-κB activation and TNFα expression. PXR activation also inhibited endotoxin-stimulated TNFα secretion from liver monocytes/macrophages isolated from PXR^+/+^ mice, but not from cells isolated from PXR^−/−^ mice. To confirm that PXR activation inhibits NF-κB *in vivo*, 3x-κB-luc fibrotic mice (which express a luciferase gene regulated by NF-κB) were imaged after treatment with the hepatotoxin CCl_4_. PXR activator inhibited the induction of hepatic NF-κB activity without affecting CCl_4_ toxicity/hepatic damage. Using a PXR reporter gene assay, cyclosporin A – but not FK506 – was shown to be a direct PXR activator, and also to induce expression of the classic PXR-regulated CYP3A4 gene in human hepatocytes and in a cell line null for the FXR, a nuclear receptor with similar properties to the PXR. *Conclusion*: PXR activation is anti-inflammatory in the liver and the effects of cyclosporin A in PBC disease recurrence may be mediated in part *via* the PXR. Since PXR activation promotes hepatocyte growth and is also anti-fibrogenic, the PXR may be an excellent drug target for the treatment of chronic inflammatory liver disease.

## 1 Introduction

The pregnane X receptor (PXR) is a member of the nuclear receptor gene superfamily of ligand-activated transcription factors expressed most prominently in hepatocytes and gut epithelium [1]. The ligand binding site of the PXR is activated by a range of structurally diverse xenobiotics such as the antibiotic rifampicin (RIF) and endobiotics (e.g. bile acids) [2]. On activation, the PXR regulates the expression of a sub-set of genes encoding drug metabolising (e.g. CYP3A) and drug transporter proteins (e.g. MRP2), that contain response elements within their promoters [3]. The canonical function of the PXR is therefore to sense elevations in xenobiotics and endobiotics and to orchestrate a response that promotes xenobiotic/endobiotic metabolism and excretion [4].

Recent work in this laboratory has revealed that the PXR has a non-canonical function that results in an inhibition in the progression of liver fibrosis [5–8]. Human fibrogenic myofibroblasts express significant levels of PXR mRNA and protein and treatment with PXR activators inhibits their trans-differentiation from hepatic stellate cells to fibrogenic myofibroblasts; inhibits myofibroblast expression of the major pro-fibrogenic cytokine TGFβ and markedly slows myofibroblast proliferation *in vitro* [6]. Using mice with a disrupted PXR gene, it was also shown that pregnenolone 16α carbonitrile (PCN), a potent activator of rodent PXR (but which only weakly activates the human PXR [9]), inhibited fibrosis *in vivo* in a PXR-dependent manner [5].

These observations may be of significant clinical value because some chronic liver disease patients are treated with PXR activators for extended periods, most notably patients with primary biliary cirrhosis (PBC). PBC is an inflammatory liver disease of unknown aetiology [10,11]. It is characterised by immune cell recruitment to the portal tracts of the liver and progressive destruction of the intra-hepatic bile ducts [10,11]. Resultant cholestasis over many years leads to chronic liver damage, fibrosis and cirrhosis. There is no treatment for PBC but many patients are often treated with the PXR activator RIF [12–14]. However, the rationale for its use is that it reduces pruritus [10,11].

A major regulator of inflammation is the transcription factor NF-κB which regulates the expression of a diverse array of genes associated with both innate and adaptive immunity (including many cytokines, chemokines, adhesion proteins and stress response genes [15]). Recently, it has been shown that the p65 sub-unit of NF-κB interacts with the PXR dimerisation partner RXRα, and disrupts binding to promoters [16]. This interaction may account for the inhibition in drug metabolism by inflammation [17]. However, it is now clear that the interaction of NF-κB with the PXR has a reciprocal inhibitory effect on NF-κB activity [18]. This has been clearly documented in the gut, which has increased NF-κB target gene expression in PXR null mice [18]. This may be of relevance to humans since PXR polymorphisms have been linked to increased inflammation of the bowel in man [19]. More recently, it has been shown that PCN (a rodent-specific activator of the PXR) inhibits the expression of several – but not all – NF-κB-dependent inflammatory genes in a mouse model of inflammatory bowel disease, an effect lost in PXR^−/−^ mice [20].

We demonstrate in this paper that feeding the PXR activator PCN to SJL/J-PXR^+/+^ mice reduced levels of the pro-inflammatory cytokines TNFα and Il-1α in the liver and the number of inflammatory cells around the portal tracts. These effects of PXR activator were lost in PXR^−/−^ mice. We show that PXR was expressed in inflammatory cells; that PXR activators inhibited NF-κB activity and that hepatic NF-κB is inhibited by PXR activation *in vivo* using live whole body imaging. Finally, we demonstrate that cyclosporin A (CsA) is a PXR activator, which may account for the beneficial effects of this anti-rejection drug (which delays disease recurrence in transplant recipients with PBC) compared to FK506 [21,22].

## 2 Materials and methods

### 2.1 Materials

d-Luciferin was obtained from Synchem (Altenburg, Germany). Recombinant human tumour necrosis factor-α (TNFα) and hyperforin (HYP) were purchased from Calbiochem (Nottingham, UK). CCl_4_, PCN, endotoxin/lipopolysaccharide (LPS) from *E. coli* 0111:B4, RIF, metyrapone (METYR), CsA and FK506 were purchased from the Sigma Chem Co. (Poole, UK). The IκB kinase 2 inhibitor (IKK2-In) was a generous gift from Wyeth Research. All other reagents were of the highest purity available from local commercial sources. Note that there are important species differences in the ligands that activate PXR. The classic rodent PXR activator PCN is a weak activator of the human PXR [4]. Accordingly, PCN has been used primarily as an activator in studies with mice whereas the classic human PXR activator RIF and other activators have been used in studies with human cells.

### 2.2 Animals

Wild type SJL/J mice initially supplied by Charles River (Margate, UK) were crossed with C57Bl6 PXR^−/−^ mice (originally obtained from Dr Steven Kliewer, University of Texas, USA [23]) to generate heterozygous (PXR^+/−^) animals. These mice were then back-crossed 6 times with wild type SJL/J mice to produce PXR^+/−^ mice on an SJL/J background and then crossed to generate SJL/J-PXR^+/+^ and SJL-PXR^−/−^ colonies. Mice were routinely genotyped by two separate PCR reactions to detect wild type and knockout alleles in DNA derived from tail tips as outlined [5].

3x-κB*-luc* C57BL/6J × CBA/J mice (bearing a transgene composed of three NF-κB sites from the Ig κlight chain promoter coupled to the gene encoding firefly luciferase) were generated and genotyped as previously described [24].

### 2.3 Animal studies

To assess the effects of PXR activation on portal tract inflammation (PTI), female mice (which show constitutively greater levels of PTI than males and significant increases in response to treatment with the E2 sub-unit of the pyruvate dehydrogenase [25]) were acclimatized to powder diet for 1 week prior to addition of PCN to treated groups at 0.025% (w/w) for 8 weeks.

CCl_4_ was used to cause liver damage in mice (25–30 g body weight) by administration of 1.0 ml CCl_4_/kg body weight (using 1:1 (v/v) CCl_4_: corn oil mixture by *i.p*. injection twice weekly for 4 weeks. Control animals received equivalent vehicle injections. PCN was co-administered at 100 mg/kg body weight as a suspension in the CCl_4_/vehicle or vehicle alone 24 h prior to endpoints only.

### 2.4 RT-PCR

Total RNA was purified from cells using Trizol (Invitrogen, Paisley, UK) and DNase I (Promega, Southampton, UK) treated to remove any contaminating genomic DNA. RT-PCR was performed and analysed essentially as previously outlined with gene specific primer sequences (Supplementary Table 1) [6]. Quantitative analysis of specific mRNA expression was performed by real-time qRT-PCR using SYBR green chemistry (or Applied Biosystem Taqman Hs00243666-m1, which detects all 3 human PXR mRNA transcripts) and an Applied Biosystems 7500 Fast Real-Time PCR System (Perkin Elmer-Applied Biosystems, Foster City, CA). Relative differences in target gene mRNA expression were assessed for each sample, in triplicate, along with appropriate no template controls after normalization of the total amount of tested cDNA to an 18S rRNA endogenous reference (Cat #: 4308329, Perkin Elmer-Applied Biosystems, Foster City, CA).

### 2.5 Pathology

Livers were excised, divided and fixed in formalin or snap frozen in liquid nitrogen and stored at −80 °C for later analysis. Formalin-fixed liver was embedded in wax and sections stained with haemotoxylin and eosin [5]. PTI was determined in at least 10 randomly selected portal tracts per animal by an examiner blinded to the treatment groups. Another blinded examiner then calculated the total area of bile duct present in each field of view. The two sets of data were then combined to generate an inflammatory score normalised to bile duct area.

### 2.6 Cell culture

The human monocytic cell lines THP-1 and U937-3XκB-LUC (stably transfected with an NF-κB responsive luciferase reporter construct [26]) were cultured in RPMI supplemented with 10% FCS, 80 μg/ml penicillin and 80 μg/ml streptomycin. The LX-2 (stellate) and Hep-G2 (hepatoma) human cell lines were cultured as previously outlined [6]. The human colon cancer cell line LS180 was obtained from Prof Ann Daly (Newcastle University, UK) and was cultured in the same manner as Hep-G2 cells. Human Kupffer cells were isolated from the non parenchymal cell pellet obtained after density gradient centrifugation with Optiprep (Sigma) during isolations for hepatic stellate cells as outlined [6], then through their rapid adherence to cell culture plastic for 10 min at 37 °C. Extensive washing with Hank's balanced salt solution (HBSS, 137 mM NaCl, 5.4 mM KCl, 0.44 mM KH_2_PO_4_, 0.34 mM Na_2_HPO_4_, 5.6 mM glucose, 6 mM HEPES, 4.1 mM NaHCO_3_ and 30 μM phenol red pH 7.4) resulted in >90% Kupffer cells as judged by immunocytochemical analyses and the uptake of latex beads [27]. Kupffer cells were cultured in serum-free RPMI medium (Invitrogen) supplemented with 80 μg/ml penicillin, 80 μg/ml streptomycin and 32 μg/ml gentamycin for 2 h prior to experimental use. Cell viability was determined by either mixing cell suspensions 1:1 (v/v) with 0.4% (w/v) trypan blue and assessing the percentage number of cells excluding the dye within 5 min, or by using the ([3-(4,5-dimethylthiazol-2-yl)-5-(3-carboxymethoxyphenyl)-2-(4-sulfophenyl)-2H-tetrazolium salt (MTS) assay (Promega, Southampton, UK). In brief, cells were incubated for 3 h with the MTS dye essentially as outlined by the manufacturers and then dye reduction determined by measuring absorbance at 490 nm using a 96-well plate reader. The use of human liver cells for scientific research was approved by the Grampian Regional Ethics and Newcastle and North Tyneside Local Regional Ethical Committee 2 and was subject to informed patient consent. Up to 10 mice for each genotype were used for isolation of murine Kupffer cells. Cell genotype was checked by PCR as outlined [5] to confirm cells were all of the correct genotype. Mouse cells were cultured as outlined for human Kupffer cells.

### 2.7 Immunocytochemistry

Cells were fixed and immunostained essentially as described [6] using either an anti-ED1 (CD68) mouse monoclonal (Serotec); an anti-β-actin mouse monoclonal (Chemicon, Chandlers Ford, UK); or a rabbit polyclonal anti-CD115 serum (Abcam, Cambridge, UK). Rhodamine red-conjugated anti-mouse IgG (Jackson) and/or FITC-conjugated anti-rabbit IgG (Abcam, Cambridge, UK) incubation was followed by fluorescence microscopy to visualise stained cells. Cells stained without the addition of primary antibody acted as controls, with control photomicrographs obtained under identical illumination and exposure conditions.

### 2.8 Transfection and reporter gene assays

THP-1 cells were transfected by electroporation using a Biorad Gene Pulser. Briefly, 5 × 10^6^ cells were washed twice in serum-free medium and re-suspended in 700 μl of serum-free medium containing a total of 40 μg of plasmid and incubated on ice for 10 min. The cells were then subjected to a single pulse at 0.23 kV, 960 μf, incubated on ice for 10 min followed by 10 min at room temperature prior to dilution into 12 ml of serum-containing medium. LX-2 cells were transfected using genejuice (Calbiochem, Loughborough, UK) essentially as previously outlined [6]. TNFα-Prom (containing 1260 nucleotides of the mouse proximal TNFα promoter inserted into the pXP1 vector [28]) and the empty pXP1 vector were originally obtained from Prof Peter Johnson (NCI, Frederick, USA). (ER6)_3_-pGL3promoter was originally described for the determining PXR activation [6]. In all cases, cells were co-transfected with a control renilla reporter construct (RL-TK, Promega, Southampton, UK). Reporter gene expression was assayed by luminometry using a Dual Luc kit (Promega, Southampton, UK) and luciferase expression normalised to renilla expression. Luciferase expression in U937-3XκB-LUC cells stably transfected with luciferase was determined also using the Dual Luc kit.

### 2.9 FACS analysis

Fluorescence activated cell sorting was used to analyse populations of cells in the sub-G1 phase of the cell cycle. Briefly, U937-3XκB-LUC cells were washed twice with PBS by centrifugation at 500 × *g* (3 min at 25 °C) before re-suspension in hypotonic fluorochrome solution (50 μg/ml propidium iodide, 0.1% (w/v) sodium citrate and 0.1% (v/v) Triton X-100). FACS analysis was carried out using a BD Facscan Flow Cytometer (Becton Dickinson, UK) and data analysis with CellQuest™ Pro Software (Becton Dickinson, UK).

### 2.10 Imaging

An IVIS ultrasensitive camera (Xenogen) was used to Image 3x-κB*-luc* mice essentially as previously outlined [29]. Mice were anesthetized with isoflurane and hair removed from the abdominal region by shaving. d-Luciferin (120 mg/kg) dissolved in 200 μl PBS, pH 7.8, was injected *i.p*. and mice were placed in the IVIS imaging chamber and a luminescence reading collected (for 2 min) 10 min later. Mice were then killed by cervical dislocation, blood was collected for serum analysis and organs removed for imaging (performed 35 min after injection of luciferin). All images were processed with the software Image-Pro Plus 4.0 (Media Cybernetics, Silver Spring, MD) integrated with the HPD-LIS module developed by Hamamatsu.

## 3 Results

### 3.1 PXR activation inhibits inflammation in an animal model of PTI

**Fig. 1:**
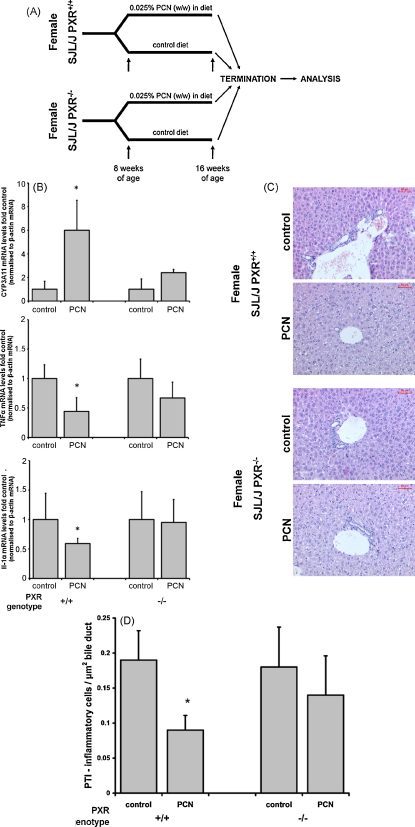
PCN inhibits PTI in SJL/J mice via the PXR. (A) Schematic diagram of the experimental approach employed to examine the effects of PXR activation on PTI. (B) Quantitative RT-PCR analysis for the mRNA transcript indicated. Data expressed relative to its equivalent genotype control. *Significantly different (*P* > 95%) transcript level versus control group using Student's *t*-test (two tailed). (C) Typical views of H&E stained liver sections from treatment groups as indicated. (D) Quantitation of the number of inflammatory cells present in portal tracts. At least 10 portal tracts from each animal were randomly selected by an examiner blinded to the treatment and the number of inflammatory cells determined. A separate blinded examiner determined the total area of bile duct in each field of view. The mean PTI for each animal was calculated and data are expressed as the mean and standard deviation PTI for 4 animals per treatment group. Total area and number of bile ducts between groups (data not included) did not significantly differ between treatment groups, suggesting that quantitation is not biased to size of portal regions of the liver. *Significantly different (*P* > 95%) number cells versus control group using Student's *t*-test (two tailed).

The SJL/J mouse strain is resistant to the induction of immune tolerance [30,31], is susceptible to cell sarcomas [32] and has been used as a model for several autoimmune diseases [33–36]. PTI and cholangitis is also observed in this strain of mice [37,38]. To determine whether PXR activation affects PTI, female PXR^+/+^ and PXR^−/−^ mice were generated on an SJL/J background (SJL/J-PXR^+/+^ and SJL/J-PXR^−/−^) and treated between 8 and 16 weeks of age with the rodent-specific PXR activator PCN (Fig. 1A). Fig. 1B confirms that there was an induction of the PXR-inducible cyp3a11 mRNA [1,23], indicating that mice were exposed to sufficient levels of PCN to activate the PXR and bring about a change in hepatic PXR-dependent gene expression in mice with a functional PXR. The lack of induction of cyp3a11 in SJL/J-PXR^−/−^ confirms functional knockout of the PXR gene in these mice [23]. Fig. 1B also shows that PCN treatment significantly inhibited the expression of TNFα and Il-1α mRNA expression in SJL/J-PXR^+/+^ mice, but not SJL/J-PXR^−/−^ mice, demonstrating that PCN reduced expression of these pro-inflammatory cytokines *via* the PXR. A statistically non-significant reduction in hepatic TNFα mRNA was observed in PCN-treated SJL/J-PXR^−/−^ mice, which may have become significantly lower (though less so than in PCN-treated SJL/J-PXR^+/+^ mice) if the numbers of animals in each group were larger. This may be associated with a PXR-independent effect of PCN as previously observed in fibrosis models caused by CCl_4_ [5]. The target for PCN mediating this PXR-independent effect remains unidentified.

Other cytokines—Il-1β, Il-6 and Il-10 were not significantly affected by PCN treatment (data not shown). Fig. 1C and D demonstrates that PXR activation in these animals also had a functional effect on hepatic inflammation since PTI was significantly reduced in SJL/J-PXR^+/+^ – but not SJL/J-PXR^−/−^ – mice, by PCN treatment. These data therefore indicate that the PXR mediates an anti-inflammatory effect of PCN *in vivo*.

### 3.2 The activated PXR inhibits NF-κB and TNFα expression in monocytic cells

**Supplementary Fig. 1:**
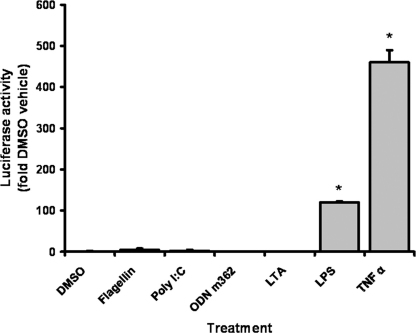
U937-3XκB-LUC cells respond to LPS and TNFα, but not to several other toll-like receptor ligands. Luciferase activity after 5 h treatment with the indicated compounds (DMSO, 0.5% (v/v); Flagellin, 10 ng/ml; poly I:C, 25 ng/ml; ODN m362, 5 μM oligodeoxynucleotide m362; LTA, 2 μg/ml lipoteichoic acid; LPS, 300 ng/ml; TNFα, 10 ng/ml. Data are the mean and standard deviation of 3 determinations from the same experiment, typical of at least 3 separate experiments. *Significantly different (*P* > 95%) reporter gene expression versus TNFα-induced DMSO control using Student's *t*-test (two tailed).

**Supplementary Fig. 2:**
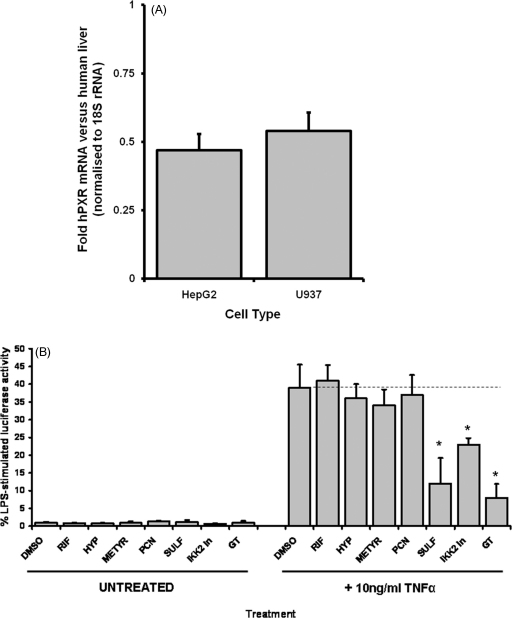
PXR activation does not inhibit TNFα-induced luciferase activity in U937-3XκB-LUC cells. (A) quantitative RT-PCR for PXR mRNA, levels compared to human liver (NHL5) using Taqman (Applied Biosystems) amplification. (B) Luciferase activity after 5 h treatment with the indicated compounds (DMSO, 0.5% (v/v); RIF, 20 μM; HYP, 1 μM; METYR, 250 μM; PCN 20 μM; SULF 1 mM; IKK2-In 10 μM), normalised to TNFα + DMSO vehicle activity. Data are the mean and standard deviation of 3 determinations from the same experiment, typical of at least 4 separate experiments. *Significantly different (*P* > 95%) reporter gene expression versus TNFα-induced DMSO control using Student's *t*-test (two tailed).

**Fig. 2:**
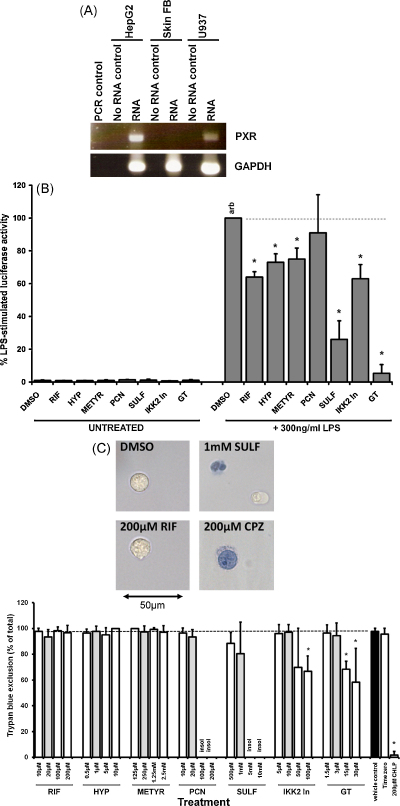
Effect of PXR activators on NF-κB-dependent gene expression in U397-3XκB-LUC cells. (A) Left panel, RT-PCR for PXR and GAPDH in U937-3XκB-LUC cells, Hep-G2 cells and human skin fibroblasts (Skin FB). Each lane is amplified product from the equivalent 50 ng template total RNA per well, 35 PCR cycles). (B) Luciferase activity after 5 h treatment with the indicated compounds (DMSO, 0.5% (v/v); RIF, 20 μM; HYP, 1 μM; METYR, 250 μM; PCN 20 μM; SULF 1 mM; IKK2-In 10 μM; GT, 3 μM) normalised to LPS + DMSO vehicle activity. Data are the mean and standard deviation of 3 determinations from the same experiment, typical of at least 4 separate experiments. *Significantly different (*P* > 95%) reporter gene expression versus LPS-induced DMSO control using Student's *t*-test (two tailed). (C) Trypan blue exclusion. Upper panels, light micrographs of typical views 5 h after treatment with 300 ng/ml LPS and the indicated compound. Lower panel, percentage trypan blue exclusion in U937-3XκB-LUC cells treated with 300 ng/ml LPS and the indicated concentration of compound for 5 h (note, grey bars are the concentrations employed to examine effects on NF-κB activity in B). Data are the mean of 3 separate incubations from the same experiment, typical of 3 separate experiments. Insol, compound became insoluble when added to culture medium. *Significantly different (*P* > 95%) trypan blue exclusion versus LPS-induced vehicle control (DMSO) using Student's *t*-test (two tailed). Similar results were obtained with compounds without the addition of LPS (data not shown).

TNFα is a potent pro-inflammatory cytokine expressed by monocytic cells. Induction of TNFα expression is regulated by the transcription factor NF-κB [15]. To determine whether PXR activation inhibits NF-κB, the human monocytic U937-3XκB-LUC cell line was treated with the toll-like receptor 4 (TLR4) ligand bacterial LPS to activate NF-κB (these cells only responded to LPS and TNFα amongst a range of other activators examined—see Supplementary Fig. 1). Fig. 2A demonstrates that the U937-3XκB-LUC cell line expressed the PXR mRNA (quantitatively, both Hep-G2 and U937-3XκB-LUC cells express lower but significant (∼50%) levels of PXR mRNA when compared to human liver—Supplementary Fig. 2).

Fig. 2B shows that co-treating cells with LPS and IKK inhibitors – sulfasalazine (SULF) and IKK2-In – or the 20S proteasomal inhibitor gliotoxin (GT) inhibited luciferase expression induced by LPS (and TNFα—Supplementary Fig. 2) as expected since these compounds are known to inhibit the activation of NF-κB [39,40]. However, Fig. 2B indicates that human PXR activators RIF, HYP and MET [9,41] also inhibited luciferase expression whereas the rodent-specific PXR activator PCN had no effect.

**Supplementary Fig. 3:**
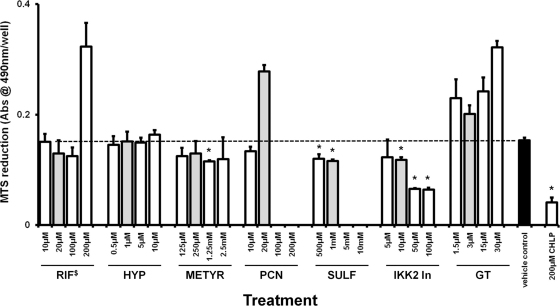
Concentrations of PXR activators used to examine effects on NF-κB activity in U937-3XκB-LUC cells are not toxic as determined by the MTS reduction assay. U937-3XκB-LUC cells were treated with 300 ng/ml LPS and the indicated concentration of compounds (prepared in 200-fold molar DMSO solvated stocks) or 0.5% (v/v) DMSO vehicle control for 5 h (note, grey bars are the concentrations employed to examine effects on NF-κB activity in Supplementary Fig. 2B). For the final 3 h, 5 μl of MTS was added to 100 μl of cell suspension in triplicate in 96-well plates prior to measurement of MTS reduction by absorbance at 490 nm in a plate reader. Data are the mean of 3 separate determinations from the same experiment, typical of 3 separate experiments. Insol, compound became insoluble when added to culture medium. *Significantly lower (*P* > 95%) MTS versus LPS-induced vehicle control (DMSO) using Student's *t*-test (two tailed). Similar results were obtained with compounds without the addition of LPS (data not shown). ^$^Compound absorbance significantly interfered with assay and subtracted from MTS using a cell free duplicate.

Fig. 2C demonstrates that none of the compounds – at the concentrations chosen to investigate effects on NF-κB activity – showed overt signs of causing toxicity with or without LPS treatment, as judged by trypan blue exclusion after 5 h of incubation. In contrast chlorpromazine, which has been shown to cause necrosis in a range of cell types [42], killed all cells by 5 h. Higher concentrations of IKK2-In and GT did show signs of toxicity, whereas none of the PXR activators caused toxicity at 10-fold the concentration employed to examine effects on NF-κB activity. These data were essentially confirmed by MTS dye reduction (Supplementary Fig. 3) and indicate that the inhibition of NF-κB by PXR activators is not associated with any effects on cell viability.

**Supplementary Fig. 4:**
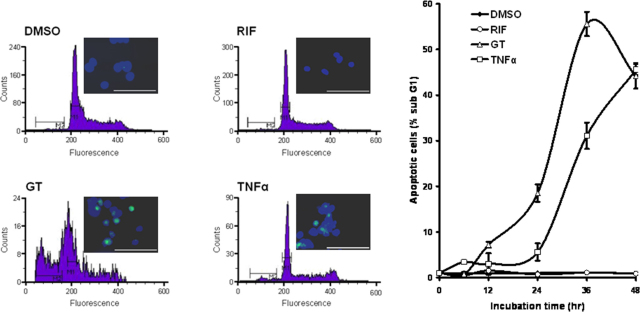
TNFα-induces U-937-3XκB-LUC cell apoptosis. FACS analysis of propidium iodide stained U-937-3XκB-LUC cells with typical active caspase 3 (green) and nucear DAPI (blue) staining [inset] after 24 h treatment with the indicated compound; right, time course for the increase in apoptotic cells using propidium iodide FACS staining. DMSO 0.1% (v/v); RIF, 20 μM; GT, 3 μM; TNFα, 10 ng/ml.

PXR activators did not inhibit TNFα-induced luciferase expression (Supplementary Fig. 2) although it should be noted that TNFα treatment stimulated U937-3XκB-LUC cell apoptosis (Supplementary Fig. 4) which may have abrogated any PXR-dependent inhibition of NF-κB.

**Fig. 3:**
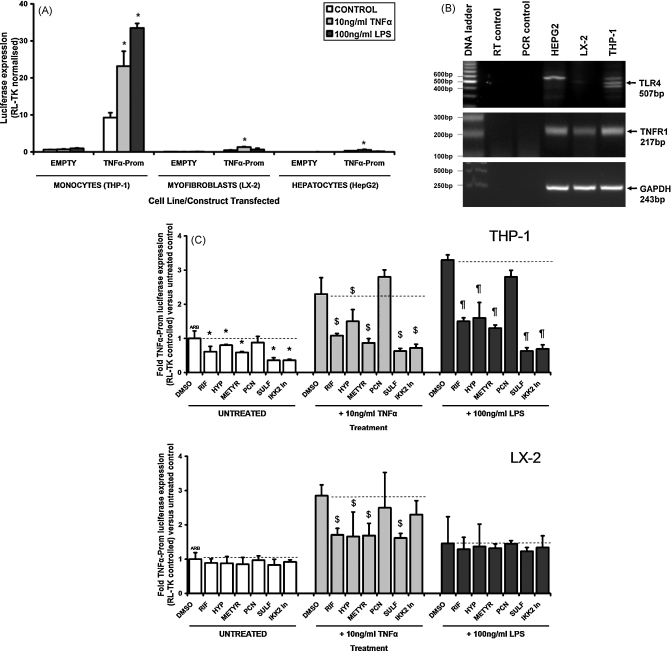

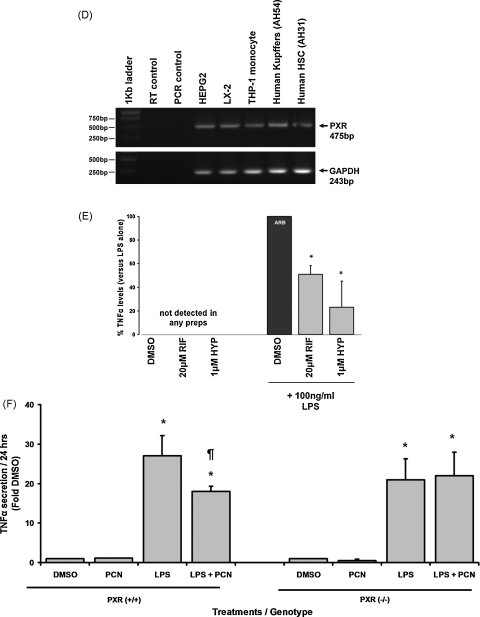
Effect of PXR activators or NF-κB inhibitors on TNFα-promoter reporter gene expression. (A and C) Cells were transfected with either pXP1 (EMPTY) or TNFα-Prom, both with renilla plasmid RL-TK (10:1 ratio). After 24 h, cells were treated as indicated (DMSO, 0.5% (v/v); RIF, 20 μM; HYP, 1 μM; METYR, 250 μM; PCN 20 μM; SULF 1 mM; IKK2-In 10 μM; GT, 3 μM) and after a further 24 h, cells were harvested and reporter gene expression determined. Data are the mean and standard deviation of 3 separate experiments, significantly different (*P* > 95%) reporter gene expression versus *untreated or DMSO vehicle control; ^$^versus TNFα-induced DMSO control or ^¶^LPS-induced DMSO control using Student's *t*-test (two tailed). (B) RT-PCR for TLR4 and TNFR1 in the various cell types (amplified product from the equivalent 50 ng template total RNA per well, 35 PCR cycles). (D) RT-PCR for human PXR and glyceraldehyde phosphate dehydrogenase (GAPDH) transcripts in the indicated cell types (amplified product from the equivalent 50 ng template total RNA per well, 35 PCR cycles). (E) percentage secretion of TNFα over 24 h by human Kupffer cells versus LPS treatment alone (100% arb). Cells were incubated with the indicated PXR activator (RIF or HYP) added from a 1000-fold molar concentrated stock in dimethyl sulfoxide (DMSO). DMSO, 0.1% (v/v) as control. Data are the mean and standard deviation of results from 3 separate patient cell preparations. *Significantly different secretion (*P* > 95%) versus LPS + DMSO using Student's *t*-test (two tailed). (F) TNFα secretion by Kupffer cells isolated from PXR^+/+^ or PXR^−/−^ mice expressed as the mean and standard deviation levels from 3 separate incubations from a single pool of cells versus DMSO treated cells. DMSO, 0.5% (v/v); PCN, 20 μM from a 4 mM stock in DMSO, LPS, 100 ng/ml. Results are typical of 3 separate experiments. Significantly different (*P* > 95%) compared to *DMSO or ^¶^LPS-treated cells using Student's *t*-test (two tailed).

To determine whether PXR activation is able to inhibit transcription of TNFα expression, the human monocytic THP-1 cell line (which was more amenable to transfection than U937-3XκB-LUC cells) was transfected with a luciferase reporter gene construct under control of the TNFα gene promoter gene sequence. Fig. 3A shows that the THP-1 cell readily trans-activated reporter gene expression in comparison to liver myofibroblasts (LX-2) or hepatocye-like (Hep-G2) cells although trans-activation was responsive to TNFα treatment in all cell types, only THP-1 cells responded to LPS, likely associated with its expression of the TLR4–see Fig. 3B. Fig. 3C demonstrates that constitutive reporter gene expression was inhibited by the human PXR activators RIF, HYP and METYR (but not the rodent-specific PXR activator PCN) and the IKK inhibitors SULF and IKK2-In in THP-1 cells (which also expressed the PXR, see Fig. 3D). This inhibitory response was essentially also observed in THP-1 cells treated with TNFα or LPS (Fig. 3C). PXR and IKK inhibitors also inhibited TNFα-induced reporter gene expression in LX-2 cells (Fig. 3C).

**Supplementary Fig. 5:**
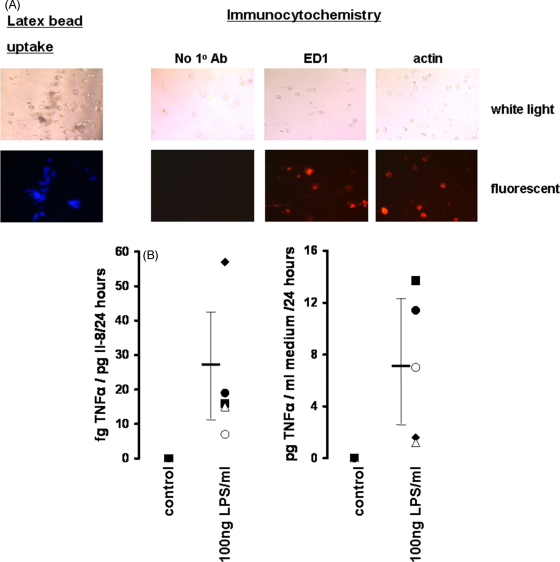
Confirmation of Kupffer cell identity. (A) Left panels, phagocyosed latex beads (5 μm diameter); right panels, immunocytochemical analysis using the indicated primary antibody. Upper panels, white light views; lower panels, fluorescent views. (B) Secretion of TNFα from human Kupffer cells isolated from different patient donors and normalised to secretion of Il-8 (only Il-8 was consistently constitutively detectable (left) (ranging between 1.6–7.2 pg day^−1^ μg cell protein^−1^) or per well (right)).

To test the relevance of these observations to inflammation and liver disease, Kupffer cells were isolated from human liver resected from 5 individuals. These cells were confirmed positive for the expression of the monocyte/macrophage marker ED1 (CD68) [43]; the uptake of latex beads [44] and expression of TNFα in response to LPS (Supplementary Fig. 5). Fig. 3D and E shows that the Kupffer cells expressed PXR mRNA and that human PXR activators inhibited LPS-induced secretion of TNFα. Fig. 3F indicates that this response is likely to be mediated *via* the PXR since LPS-dependent TNFα secretion from Kupffer cells isolated from PXR^−/−^ mice was not affected by PCN whereas PCN inhibited secretion from Kupffer cells isolated from PXR^+/+^ mice.

### 3.3 PXR activation inhibits NF-κB activation *invivo*

**Fig. 4:**
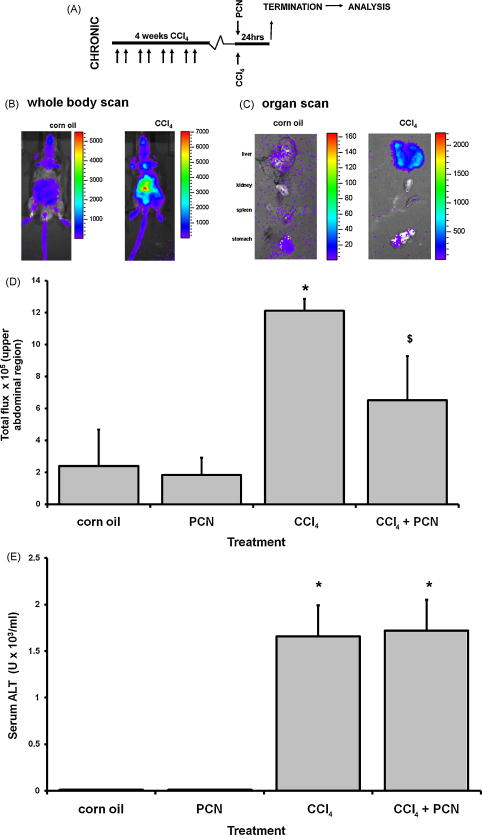
PCN inhibits hepatic NF-κB transcriptional activity induced by CCl_4_ treatment *invivo*. (A) Schematic diagram of the experimental approach employed to examine the effects of PXR activation on NF-kB. (B) Light production from a corn oil control and CCl_4_-treated mouse 24 h after treatment, 10 min after injection of d-luciferin. (C) Light production from the indicated organs removed from a corn oil or CCl_4_-treated mouse, 35 min after injection of d-luciferin. Data are typical of at least 3 animals/treatment group. (D) Total light production from the abdominal region of mice 24 h after treatment as indicated. (E) Serum ALT activity from imaged mice 24 h after treatment as indicated. Data are the mean and standard deviation of at least 3 animals per treatment group. *Significantly different from corn oil and PCN, ^$^significantly different from CCl_4_ using ANOVA statistical test.

CCl_4_ is a pro-toxin that is metabolised primarily by cytochrome P450 2e1 to a radical species which forms a reactive peroxy-radical that initiates lipid peroxidation and cell damage in the liver [8]. We have previously shown that PCN did not interfere with the metabolism or toxicity of CCl_4_ in both rats and mice and that the anti-fibrogenic effects of PCN were independent of a modulation of toxicity [5]. CCl_4_ was therefore administered to 3x-κB*-luc* C57BL/6J × CBA/J mice for 4 weeks to generate a chronic injury and inflammation. At the final injury, PCN or vehicle was administered (Fig. 4A) and the level of NF-κB activation (*via* luciferase transgene expression) determined in the liver *in vivo* 24 h later. Fig. 4B demonstrates that CCl_4_ treatment induced NF-κB activity and that light emission in whole body imaging was primarily associated with emission from the liver since nearby organs (kidney, spleen and stomach) showed markedly lower light emission than the liver after dissection (Fig. 4C). PCN treatment significantly reduced NF-κB activity in CCl_4_-treated mice (Fig. 4D) without affecting liver damage as judged by serum ALT levels (Fig. 4E) or through histological examination of fixed liver sections after H&E staining (data not shown). The lack of effect of PCN on hepatic necrosis in these studies is consistent with previous investigations and therefore suggests that PCN inhibits NF-κB by some other mechanism(s)

### 3.4 CsA is a PXR activator

**Fig. 5:**
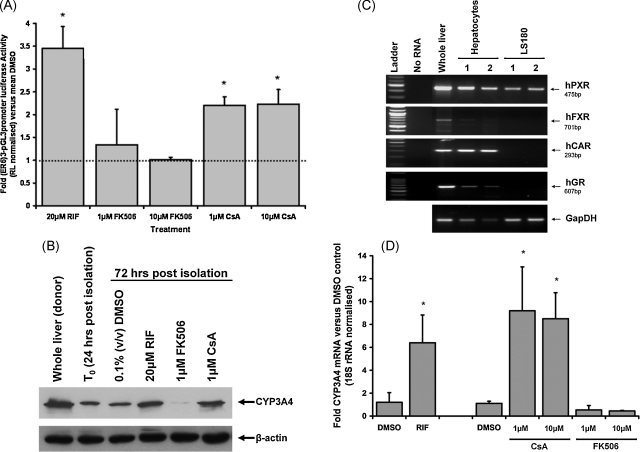
CsA is an activator of the human PXR. (A) Hep-G2 cells cultured in 24-well plates were transfected with 0.5 μg (ER6)_3_-pGL3promoter and 0.05 μg RL-TK/well and PXR activation determined [6]. Data are the mean and standard deviation of at least 3 separate transfections from the same experiment expressed as fold normalised luciferase expression versus DMSO vehicle control, typical of at least 3 separate experiments. *Significantly different (*P* > 95%) luciferase activity versus DMSO using Student's *t*-test (two tailed). (B) Human hepatocytes were isolated and cultured as outlined in Methods section and treated with the indicated compounds after the first 24 h of culture (*T*_0_) for a further 48 h prior to isolation and analysis of CYP3A4 and β-actin levels by Western blotting. Medium and treatments were replenished every 24 h. (C) RT-PCR for the indicated transcripts in human liver, two separate human hepatocyte preparations (freshly isolated cells) and two separate LS180 cultures (amplified product from the equivalent 100 ng template total RNA per well, 35 PCR cycles). (D) Expression of CYP3A4 mRNA in LS180 cells. LS180 cells were treated with the indicated compound DMSO, 0.1% (v/v); RIF, 20 μM RIF (for 48 h) and CsA and FK506 at the indicated concentrations for 72 h. Culture media and treatments were changed daily. RNA was isolated and transcript CYP3A4 and 18S rRNA levels determined by quantitative RT-PCR. *Significantly different (*P* > 95%) transcript level activity versus DMSO using Student's *t*-test (two tailed).

PBC is a chronic cholestatic inflammatory liver disease of unknown cause. Chronic PTI and fibrosis is untreatable and leads to cirrhosis and the need for liver transplantation. A recent report has determined that PBC disease re-emerges in PBC patients faster post-transplant when they were maintained on the anti-rejection drug FK506 compared to patients on CsA [21]. Fig. 5A demonstrates that CsA – but not FK506 – was a PXR activator in a human PXR trans-activation reporter gene assay. This was confirmed in human hepatocytes which classically respond to the potent PXR ligand RIF with high levels of CYP3A4 induction *via* its activation of the PXR [9]. Fig. 5B shows that CsA – but not FK506 – induced CYP3A4 protein in human hepatocytes. Since CYP3A4 is also regulated by FXR [45], a colon cell line (LS180) was tested since these cells did not express the FXR but retained PXR expression (Fig. 5C). CsA induced CYP3A4 mRNA (Fig. 5D) suggesting that CsA activates, either directly or by some intermediary mechanism, at least the PXR to mediate its effects on CYP3A4 mRNA.

## 4 Discussion

Liver damage is primarily associated with hepatocyte necrosis and an inflammatory response that is a component of the stimulus for regeneration and repair. With chronic damage, fibrosis occurs which, although considered an essential response to repair, may have a pro-inflammatory effect that in some individuals, leads to an exacerbation of disease. There is good evidence to suggest that a reduction in fibrosis may improve the liver's response to on-going insult [40,46]. Inflammation is a component of fibrosis and therefore an inhibition of inflammation may also have a similar effect. Currently there is no treatment available for chronic liver disease and liver fibrosis [8].

The PXR is a nuclear receptor that regulates the expression of the major drug metabolising enzymes in human liver, collectively referred to as the cytochrome P450 3A sub-family. These enzymes are expressed at high levels in hepatocytes. Like many cytochrome P450s, they have endogenous functions, which for CYP3A4 in the liver, is to metabolise bile acids to more water soluble/excretable products [23,47]. This canonical function of the PXR is likely to be an advantage in cholestatic liver disease since it would promote the urinary excretion of toxic bile acids [23]. Previous work suggests that there is a non-canonical anti-fibrotic role for the PXR since activators inhibited fibrosis in animal models of chronic liver disease. The myofibroblast – the major contributor to fibrosis – was shown to be particularly responsive to PXR activators in human cells *in vitro* [6]. Human myofibroblasts were shown to express high levels of PXR yet these cells did not express the genes regulated by the PXR in hepatocytes [6]. The function of the PXR in these cells is unclear, however the effects of PXR activation were striking, with RIF maintaining cells in a quiescent non-fibrogenic phenotype *in vitro* for several weeks [6]. Since these discoveries, the PXR has been shown to be anti-inflammatory in the gut [19,20] and therefore we investigated a potential anti-inflammatory role in the liver.

The data in this paper indicate that PXR activation inhibited PTI in the SJL/J mice and reduced the levels of inflammatory cytokines such as TNFα in the liver. TNFα is expressed primarily by monocytes and macrophages, suggesting that the effects observed in these experiments are likely associated with a direct effect of PXR activation in these cell types. This potential was confirmed *in vitro* using monocyte and macrophage cells. These cell types expressed the PXR, and NF-κB – the major transcriptional regulator of inflammatory gene expression – was shown to be inhibited by PXR activators. Imaging experiments confirmed PXR activation inhibited liver NF-κB activity *in vivo*.

Activation of the PXR in a chronically damaged liver may therefore have at least 4 potential benefits with regard to ameliorating the effects of chronic cholestatic liver disease. The first is the well established promotion of hepatocyte growth [48,49], an essential requirement in a liver that is losing functional capacity. The second is an increase in cytochrome P450 3A expression, which will enhance the ability of the liver to reduce toxic bile acid levels [23]. The third is an inhibition in fibrosis [5,6]. The final benefit – indicated by these experiments – is an inhibition of inflammation. All these factors impact upon each other and it is likely that the inhibition of fibrosis previously observed was dependent in part, on an effect on inflammation.

Increasing attention is being paid to the cross-talk that occurs between nuclear receptors and signalling pathways regulating inflammation. In this respect, cross-talk between the peroxiome proliferator activated receptor γ (PPARγ) and NF-κB activity has been most intensely studied. Expression of PPARγ is regulated by LPS [50] and activation of PPARγ is also anti-inflammatory [51–54] via direct protein-protein interaction with NF-κB in a DNA binding-independent mechanism [55,56]. PPARγ may also be SUMOylated, which can function to inhibit the loss of transcriptional co-repressors from NF-κB target genes [57]. Some of these mechanisms may also be involved in the PXR-dependent inhibition of inflammation.

The clinical evidence for a potential effect of PXR activators on chronic liver disease is scarce but indicative of benefit. Trials with RIF have shown that this compound reduced serum levels of alkaline phosphatase, a marker of cholestatic liver disease [58,12]. Effects on inflammation and fibrosis have not to our knowledge, been examined. RIF – although a potent activator of the human PXR – is associated with adverse hepatotoxic effects [59]. However, this is not associated with its interaction with the PXR but likely due to effects on mitochondria. Thus, other PXR activators – which do not have these latter effects – could be more effective. However, the current safe use of RIF in a group of chronic liver disease patients could be extended to use in other groups relatively rapidly and without significant financial burden.

## Conflict of interest

We here declare that there are no conflicts of interest for any of the authors or the institution.
