# Supplementary material for: The PXR is a drug target for chronic inflammatory liver disease
Source: J Steroid Biochem Mol Biol. 2010 May 31;120(2-3):137–48. doi: 10.1016/j.jsbmb.2010.04.012 (PMC2937210; doi:10.1016/j.jsbmb.2010.04.012)
Supplement: Supplementary file 1 [file mmc6.doc]

**Table 1. Oligonucleotide sequences used for RT-PCR and/or Sybr green quantification of mRNA levels.**

| Gene ID | Alternative name | Primers | Primer sequence 5’-3’ | Annealing temp ^o^C | Amplicon size (bp) | Comments |
| --- | --- | --- | --- | --- | --- | --- |
| human |  |  |  |  |  |  |
| NR1I2 | hPXR | US | acatggctgacatgtcaacctaca | 64 | 475 | Amplifies the same size amplicon from all 3 human PXR reference transcripts NM_003889, NM_022002 and NM_033013  For quantitative RT-PCR (Taqman) analysis, used Hs00243666-m1 (Applied Biosystems), which also detects all 3 transcripts. |
|  |  | DS | agctcggtgagcatagccatgat |  |  |
| NR1I3 | hCAR | US | agaacagtcagcaaaagcat | 50 | 293 | Amplifies the same size amplicon from all 15 human reference transcripts NM_001077469 to NM_001077482 and NM_005122 |
|  |  | DS | tgcacaaactgttcaaacat |  |  |
| NR1H4 | hFXR | US | tgtgtgttgtttgtggaga | 41 | 701 | Amplifies the reference transcript NM_005123 |
|  |  | DS | atcagagataccactatttc |  |  |
| NR3C1 | hGR | US | gaagcctatttttaatgtc | 42 | 607 | Amplifies the same size amplicon from all 7 human reference transcripts NM_000176, NM_001018074 - NM_001018077, NM_001020825 and NM_001024094. Note final sequence amplicon size 610bp |
|  |  | DS | atcatatcctgcatataac |  |  |
| CYP3A4 | CYP3A4 | US | tgtcctaccataagggcttttgtat | 60 | 136 | Amplifies the reference transcript NM_017460 |
|  |  | DS | ttcactagcactgttttgatcatgtc |  |  |
| GAPDH | GAPDH | US | tgacatcaagaaggtggtgaag | 55 | 243 | Amplifies the reference human transcript NM_002046. Also amplifies rat and mouse orthologues. |
|  |  | DS | tcttactccttggaggccatgt |  |  |
|  |  |  |  |  |  |  |
| TLR4 | hTLR4 | US | tggatacgtttccttataag | 56 | 507 | Amplifies the 3 reference transcripts  NR_024168, NM_138554 and NR_024169 |
|  |  | DS | gaaatggaggcaccccttc |  |  |
| TNFRSF1A | hTNFR1 | US | aggagaaacagaacaccgt | 43 | 217 | Amplifies the reference transcript NM_001065. |
|  |  | DS | ccaatgaagaggagggat |  |  |
|  |  |  |  |  |  |  |
| mouse |  |  |  |  |  |  |
| Cyp3a11 | cyp3a11 | US | gcagaaggcaaagaaagg | 55 | 170 | Amplifies the reference transcript NM_007818. |
|  |  | DS | gatcaaaaaaatcaaatc |  |  |
| Tnf | mTNFα | US | gaccaggctgtcgctacatca | 56 | 67 | Amplifies the reference transcript NM_013693. |
|  |  | DS | cgtagggcgattacagtcacgg |  |  |
| Il1a | mIl-1α | US | gacctgcagtccataacc | 55 | 172 | Amplifies reference transcript NM_010554. |
|  |  | DS | tgagatagtgtttgtcca |  |  |
| Il1b | mIl-1β | US | gggctgcttccaaacctt | 55 | 147 | Amplifies reference transcript NM_008361. |
|  |  | DS | Gggaaagacacaggtagc |  |  |
| Il6 | mIl-6 | US | ctgatgctggtgacaaccac | 51 | 143 | Amplifies reference transcript NM_031168. |
|  |  | DS | tccacgatttcccagagaac |  |  |
| Il10 | mIL-10 | US | gcctgctcttactgactg | 51 | 174 | Amplifies reference transcript NM_010548. |
|  |  | DS | ttagcagtatgttgtcca |  |  |
